# Current News

**Published:** 1903-10

**Authors:** 


### Current News.

### FOURTH INTERNATIONAL DENTAL CONGRESS.

*August 29 to September 3, 1904.*

UNIVERSAL EXPOSITION, ST. LOUIS, 1904.

*Committee of Organization of Fourth Dental Congress.*—H. J. Burkhart, Chairman; E. C. Kirk, Secretary; R. H. Hofheinz, William Carr, W. E. Boardman, V. E. Turner, J. Y. Crawford, M. F. Finley, J. W. David, William Crenshaw, Don M. Gallie, G. V. I. Brown, A. H. Peck, J. D. Patterson, B. L. Thorpe.

The Department of Congresses of the Universal Exposition, St. Louis, 1904, has nominated the Committee of Organization of the Fourth International Dental Congress—to be held in August, 1904, in connection with the Exposition—which was appointed by the National Dental Association, and has instructed the committee thus appointed to proceed with the work of organization of said Congress.

Pursuant to the instructions of the Director of Congresses of the Universal Exposition, 1904, the Committee of Organization presents for your consideration and information the subjoined outline of the plan of organization of the Dental Congress.

The Congress will be divided into two departments: Department A—SCIENCE (divided into four sections). Department B—APPLIED SCIENCE (divided into six sections).

### DEPARTMENT A—SCIENCE.

I. Anatomy, Physiology, Histology, and Microscopy. Chairman, M. H. Cryer.

II. Etiology, Pathology, and Bacteriology. Chairman, R. H. Hofheinz.

III. Chemistry and Metallurgy. Chairman, J. D. Hogden.

IV. Hygiene, Prophylaxis, Therapeutics, Materia Medica, and Electro-Therapeutics. Chairman, A. H. Peck.

### DEPARTMENT B—APPLIED SCIENCE.

V. Oral Surgery. Chairman, G. V. I. Brown.

VI. Orthodontia. Chairman, E. H. Angle.

VII Operative Dentistry. Chairman, C. N. Johnson.

VIII. Prosthesis. Chairman, C. R. Turner.

IX. Education, Nomenclature, Literature, and History. Chairman, T. W. Brophy.

X. Legislation. Chairman, William Carr.

### COMMITTEES.

The following committees were appointed:

*Finance.*—Chairman, C. S. Butler.

*Programme.*—Chairman, A. H. Peck.

*Exhibits.*—Chairman, D. M. Gallie.

*Transportation.*—(To be appointed.)

*Reception.*—Chairman, B. Holly Smith.

*Registration.*—Chairman, B. L. Thorpe.

*Printing and Publication.*—Chairman, W. E. Boardman.

*Conference with State and Local Dental Societies.*—Chairman, J. A. Libbey.

*Dental Legislation.*—Chairman, William Carr.

*Auditing.*—(Committee of Organization.)

*Invitation.*—Chairman, L. G. Noel.

*Membership.*—Chairman, J. D. Patterson.

*Educational Methods.*—Chairman, T. W. Brophy.

*Oral Surgery.*—Chairman, G. V. I. Brown.

*Prosthetic Dentistry.*—Chairman, C. R. Turner.

*Local Committee of Arrangements.*—(To be appointed.)

*Essays.*—Chairman, Wilbur F. Litch.

*History of Dentistry.*—Chairman, William H. Trueman.

*Nomenclature.*—Chairman, S. W. Foster.

*Promotion of Appointment of Dental Surgeons in the Armies and Navies of the World.*—Chairman, Williams Donnally.

*Care of the Teeth of the Poor.*—Chairman, Thomas Fillebrown.

*Etiology, Pathology, and Bacteriology.*—Chairman, R. H. Hofheinz.

*Prize Essays.*—Chairman, James Truman.

*Oral Hygiene, Prophylaxis, Materia Medica, Therapeutics, and Electro-Therapeutics.*—Chairman, A. H. Peck.

*Operative Dentistry.*—Chairman, C. N. Johnson.

*Resolutions.*—Chairman, J. Y. Crawford.

*Clinics.*—Chairman, C. E. Bentley.

*Nominations.*—(To be appointed.)

*Local Reception Committee.*—(To be appointed.)

*Ad interim.*—Chairman, G. V. I. Brown.

The officers of the Congress, president, vice-presidents, secretary, and treasurer, will be elected by the Congress at large at the time of the meeting, and will be nominated for the several positions by the nominating committee.

The Fourth International Dental Congress, which will be held August 29 to September 3, inclusive, 1904, will be representative of the existing status of dentistry throughout the world. It is intended further that the Congress shall set forth the history and material progress of dentistry from its crude beginnings through its several developmental stages, up to its present condition as a scientific profession.

The International Dental Congress is but one of the large number of congresses to be held during the period of the Louisiana Purchase Exposition, and these in their entirety are intended to exhibit the intellectual progress of the world, as the Exposition will set forth the material progress which has taken place since the Columbian Exposition in 1893.

It is important that each member of the dental profession in America regard this effort to hold an International Dental Congress as a matter in which he has an individual interest, and one which he is under obligation to personally help towards a successful issue. The dental profession of America has not only its own professional record to maintain with a just pride, but, as it is called upon to act the part of host in a gathering of our colleagues from all parts of the world, it has to sustain the reputation of American hospitality as well.

The Committee of Organization appeals earnestly to each member of the profession to do his part in making the Congress a success. Later bulletins will be issued setting forth the personnel of the organization and other particulars, when the details have been more fully arranged.

H. J. Burkhart, *Chairman.*
E. C. Kirk, *Secretary.*

Approved:
Howard J. Rogers,
*Director of Congresses.*
David R. Francis,
*President of the Exposition.*

---

### DENTAL COMMISSIONERS OF CONNECTICUT.

The Dental Commissioners of the State of Connecticut hereby give notice that they will meet at Hartford, on Wednesday, Thursday, and Friday, November 18, 19, and 20, 1903, respectively, to examine applicants for license to practise dentistry, and for the transaction of any other proper business.

The practical examination in operative and prosthetic dentistry will be held Wednesday, November 18, at 9 A.M., in Putnam Phalanx Armory, corner Haynes and Pearl Streets.

The written theoretic examination will be held Thursday and Friday, November 19 and 20, at the Capitol.

All applicants should apply to the Recorder for proper blanks, and for the revised rules for conducting the examinations.

Application blanks must be carefully filled in and sworn to, and with fee, twenty-five dollars ($25.00), filed with the Recorder on or before November 10, 1903.

By direction of the Dental Commissioners.

J. Tenney Barker,
*Recorder.*

---

### INSTITUTE OF DENTAL PEDAGOGICS.

The next annual meeting of the Institute of Dental Pedagogics will be held at Buffalo, N. Y., December 28, 29, and 30, 1903. A complete programme is being arranged which will be exceedingly interesting. It will be published in full in the next issue of this journal.

W. H. Whitslar,
*Chairman.*

---

### NATIONAL ASSOCIATION OF DENTAL EXAMINERS.

The following officers were elected at the annual meeting held at Asheville, July, 1903:

President, Burton Lee Thorpe, St. Louis, Mo.; First Vice-President, from the West, James J. Reid, Chicago, Ill.; Second Vice-President, from the East, J. Tenny Barker, Wallingford, Conn.; Third Vice-President, from the South, T. B. Whitney, Salem, Ala.; Secretary and Treasurer, Charles A. Meeker, 29 Fulton Street, Newark, N. J.

On account of the resignation of Burton L. Thorpe, D.D.S., president of this Association, James J. Reid, D.D.S., of 1204 Trude Building, Chicago, Ill., will, from September 14, assume the duties of the president.

It is earnestly requested that all the secretaries of the boards of examiners throughout the States and Territories mail to the secretary all changes in their respective boards and greatly oblige,

CHARLES A. MEEKER, D.D.S.,
*Secretary.*

29 FULTON STREET, NEWARK, N. J.

---

### NORTHEASTERN DENTAL ASSOCIATION.

THE ninth annual meeting of the Northeastern Dental Association will be held in the New Horticultural Hall, corner of Massachusetts and Huntington Avenues, Boston, Mass., October 21, 22, and 23, 1903.

An interesting and profitable meeting, with a full line of exhibits, is promised.

Boston is an ideal place for a large meeting. Please come and help make it so.

EDGAR O. KINSMAN,

*Secretary.*

CAMBRIDGE, MASS.

---

### SEVENTH AND EIGHTH DISTRICT DENTAL SOCIETIES OF NEW YORK.

THE thirty-fifth annual union meeting of the Seventh and Eighth District Dental Societies of the State of New York will be held at the Osborn House, Rochester, N. Y., October 27, 28, and 29, 1903. A most excellent meeting, with numerous clinics, is promised. One day will be devoted exclusively to clinics, with discussions of same in the evening. Application has been made for reduced railroad rates. Exhibitors desiring space are requested to communicate with the hotel or the Business Committee.

W. W. SMITH,
*Chairman Business Committee.*

